# Personal

**Published:** 1895-09

**Authors:** 


### Personal.

The following named physicians, residing in Buffalo, in some instances accompanied by their wives as noted, have recently returned or are returning from foreign travel: Dr. and Mrs. L. F. Harvey, from a tour to the Sandwich Islands, Japan, and so around the world; Dr. and Mrs. Herman Mynter, from Denmark; Dr. D. W. Harrington, from a tour in Europe; Dr. Geo. F. Cott, from a sojourn in Vienna for professional advancement; Dr. and Mrs. H. Y. Grant, from a vacation tour to the Continent, and Dr. and Mrs. A. W. Hurd, from a wedding journey abroad.

---

Thomas H. Norton, Ph. D. (Heidelberg), professor of chemistry in the University of Cincinnati, has received the honorary degree of Doctor of Science from Hamilton College. This degree is one of some rarity and has been conferred but once before in the history of the college.

---

Dr. A. L. Hummel, of Philadelphia, the eminent medical journal advertiser, has removed his business to New York and established his offices at 108 Fulton street. We have no doubt this will prove an advantageous change to the ever increasing business of this house.

---

Dr. Frank Parsons Norbury, of St. Louis, editor of the *Medical Fortnightly*, has been elected professor of medicine and clinical medicine in the College of Physicians and Surgeons.

---

Dr. J. G. Meidenbauer, of Buffalo, has removed his office from 204 High street to 291 Maple street, corner of High. Hours: 7 to 8 A. M.; 1 to 2 and 6 to 7 P. M.

---

Dr. Albert T. Lytle, of Buffalo, has lately occupied his fine new residence on the corner of Elmwood and Lexington avenue.

